# High glucose alters the DNA methylation pattern of neurodevelopment associated genes in human neural progenitor cells in vitro

**DOI:** 10.1038/s41598-020-72485-7

**Published:** 2020-09-24

**Authors:** Deepika Kandilya, Sukanya Shyamasundar, Dhiraj Kumar Singh, Avijit Banik, Manoor Prakash Hande, Walter Stünkel, Yap Seng Chong, S. Thameem Dheen

**Affiliations:** 1 Department of Anatomy, Yong Loo Lin School of Medicine, National University of Singapore, 4 Medical Drive, MD10, Level 4, Singapore, 117594 Singapore; 2 Department of Physiology, Yong Loo Lin School of Medicine, National University of Singapore, Singapore, Singapore; 3 Department of Obstetrics and Gynaecology, Yong Loo Lin School of Medicine, National University of Singapore, Singapore, Singapore; 4 Present Address: Experimental Drug Development Centre, Agency for Science, Technology and Research, Singapore, Singapore

**Keywords:** Developmental biology, Molecular biology, Neuroscience

## Abstract

Maternal diabetes alters the global epigenetic mechanisms and expression of genes involved in neural tube development in mouse embryos. Since DNA methylation is a critical epigenetic mechanism that regulates gene functions, gene-specific DNA methylation alterations were estimated in human neural progenitor cells (hNPCs) exposed to high glucose (HG) in the present study. The DNA methylation pattern of genes involved in several signalling pathways including axon guidance (SLIT1-ROBO2 pathway), and Hippo pathway (YAP and TAZ) was altered in hNPCs exposed to HG. The expression levels of SLIT1-ROBO2 pathways genes (including its effectors, SRGAP1 and CDC42) which mediates diverse cellular processes such as proliferation, neurogenesis and axon guidance, and Hippo pathway genes (YAP and TAZ) which regulates proliferation, stemness, differentiation and organ size were downregulated in hNPCs exposed to HG. A recent report suggests a possible cross-talk between SLIT1-ROBO2 and TAZ via CDC42, a mediator of actin dynamics. Consistent with this, *SLIT1* knockdown downregulated the expression of its effectors and TAZ in hNPCs, suggesting that HG perturbs the cross-talk between SLIT1-ROBO2 and TAZ in hNPCs. Overall, this study demonstrates that HG epigenetically alters the SLIT1-ROBO2 and Hippo signalling pathways in hNPCs, forming the basis for neurodevelopmental disorders in offspring of diabetic pregnancy.

## Introduction

Maternal factors such as nutrition and metabolic conditions influence the normal course of fetal development, and any disturbance in those factors may result in long term consequences in the offspring^1^. Diabetes during pregnancy leads to spontaneous abortions, stillbirths, and birth-related anomalies of various organ systems including the central nervous system (CNS)^2–4^. Neurulation involves the formation of the neural tube, which primarily consists of neural stem cells (NSCs) that sequentially undergo proliferation, differentiation, migration and lineage specification to form the CNS. These processes are regulated by several molecular signalling pathways including axon guidance and hippo signalling^5^ and dynamic changes in the epigenome^6^. Previously, we have shown that maternal diabetes perturbs the brain patterning in mouse embryos^7^. Furthermore, gene expression profiling in cranial neural tube of embryos from diabetic pregnancy showed altered expression pattern of > 300 genes involved in several functional pathways including cell proliferation, migration, and differentiation^8,9^. Recent evidence suggest that epigenetic mechanisms such as DNA methylation, histone modifications and microRNAs are key modulators of gene expression^10^, and crucial for normal brain development and neuronal function^11^. Given that maternal diabetes alters the transcriptome in the developing brain, there is a critical need to understand the epigenetic mechanisms which influence transcriptional regulation and predispose the offspring to neurodevelopmental disorders.

DNA methylation changes are dynamic during prenatal development, and aberrant DNA methylation has been shown to underlie several congenital defects including neural tube defects^12–14^. DNA methylation which primarily occurs on a CpG nucleotide, regulates transcription without altering the DNA sequence^15^. Previous studies from our lab have shown that HG/hyperglycemia altered the chromatin organisation, global DNA methylation, global histone modification and miRNA regulation in mouse embryonic neural stem cells^4,16^. Changes in epigenetic mechanisms such as DNA methylation are reversible in nature, and create a major translational impact since they are suitable for therapeutic intervention^17^.

Overall, it is apparent that understanding epigenetic mechanisms is essential for our comprehension of gene regulation during neurodevelopment. Although several studies demonstrate that HG or hyperglycemia alters the epigenetic mechanisms of genes involved in neural tube formation, comprehensive high throughput analysis is required to precisely detect the mechanistic link between the effects of maternal high glucose and development of neural tube defects in the fetus. In view of this, the present study was aimed at addressing the hypothesis that high glucose alters the gene-specific DNA methylation status of key genes that regulate neurodevelopmental processes.

In the present study, DNA methylation pattern of genes involved in several signalling pathways including axon guidance (SLIT1-ROBO2 signalling pathway)^18–21^ and Hippo signalling pathway^22–24^ contributing to brain development was found to be altered in hNPCs exposed to HG. The SLIT1-ROBO2 signalling pathway genes including SLIT1, and its effector genes ROBO2, SRGAP1 and CDC42, mediate diverse cellular processes such as proliferation, migration, neurogenesis and axon guidance, while Hippo signalling pathway effectors i.e., YAP and TAZ regulate cell proliferation, stemness, differentiation and organ size. Recently, it was reported that CDC42, one of the SLIT1 effectors and a mediator of actin dynamics, regulates Hippo signalling pathway^25^, suggesting a possible cross-talk between SLIT1-ROBO2 signalling and Hippo pathways during brain development. This study demonstrates that HG epigenetically regulates the crosstalk between SLIT1-ROBO2 and Hippo signalling pathway in hNPCs, forming the basis for neurodevelopmental disorders in offspring of diabetic pregnancy.

## Results

### Characterisation of human neural progenitor cells (hNPCs)

**Figure 1:**
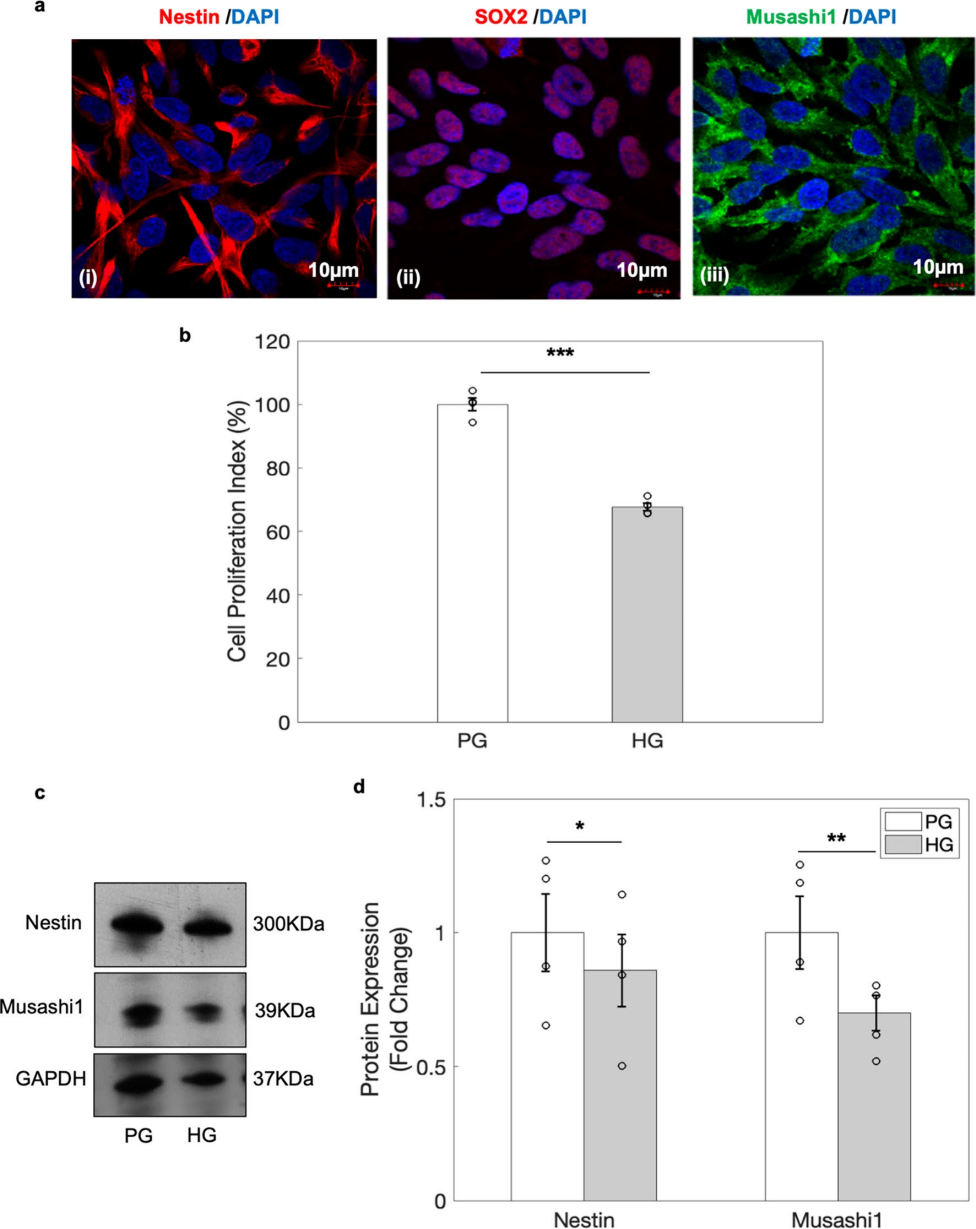
Cell proliferation was decreased in hNPCs exposed to high glucose. (**a**) Representative images of hNPCs expressing neural stem cell markers i.e., Nestin (i) SOX2 (ii), and Musashi1 (iii). Nuclei are stained with DAPI (blue). (**b**) There was significant decrease in cell proliferation index in hNPCs exposed to high glucose as depicted by MTS assay in n = 4, ***p < 0.001. (**c**) Western blot analysis revealed decrease expression of Nestin and Musashi1 in hNPCs exposed to HG for 96 h. (**d**) Quantitative analysis shows significant decrease in Nestin (Observed band size 300 kDa, Cat. no. ab18102, Abcam) and Musashi1 (39 kDa) protein expression in hNPCs exposed to HG for 96 h. Data is represented as Mean ± SE, n = 4, *p < 0.05, **p < 0.01. Uncropped blot images are shown in Supplementary Fig. S2.

Human neural progenitor cells (hNPCs) were characterised using neural stem cell markers including Nestin (*NES),* an intermediate filament protein expressed in stem cells, Sex determining region Y-Box2 (*SOX2*), a transcription factor that regulates cell fate specification of NPCs during neural tube development^26,27^ and Musashi1 (*MSI1*), a RNA binding protein that coordinates NSC self-renewal. The results showed that hNPCs used in this study expressed all the above neural stem cell markers (Fig. 1a).

### High glucose decreases the proliferation and stemness of hNPCs

In order to examine the effects of high glucose (HG) on cell proliferation, MTS assay was performed in hNPCs exposed to physiological glucose (PG, 5 mM), or High glucose (HG, 40 mM) concentration for 96 h. The cell proliferation index was found to be significantly decreased in hNPCs exposed to HG, suggesting that the expression of genes associated with cell proliferation may be deregulated by HG (Fig. 1b). Further, there was a significant decrease in the expression of stemness markers, Nestin and Musashi1 in hNPCs exposed to HG (Fig. 1c,d and uncropped blots in Supplementary Fig. S2) suggesting that HG perturbs stemness in hNPCs.

### High glucose alters the genome-wide DNA methylation status in hNPCs

**Figure 2:**
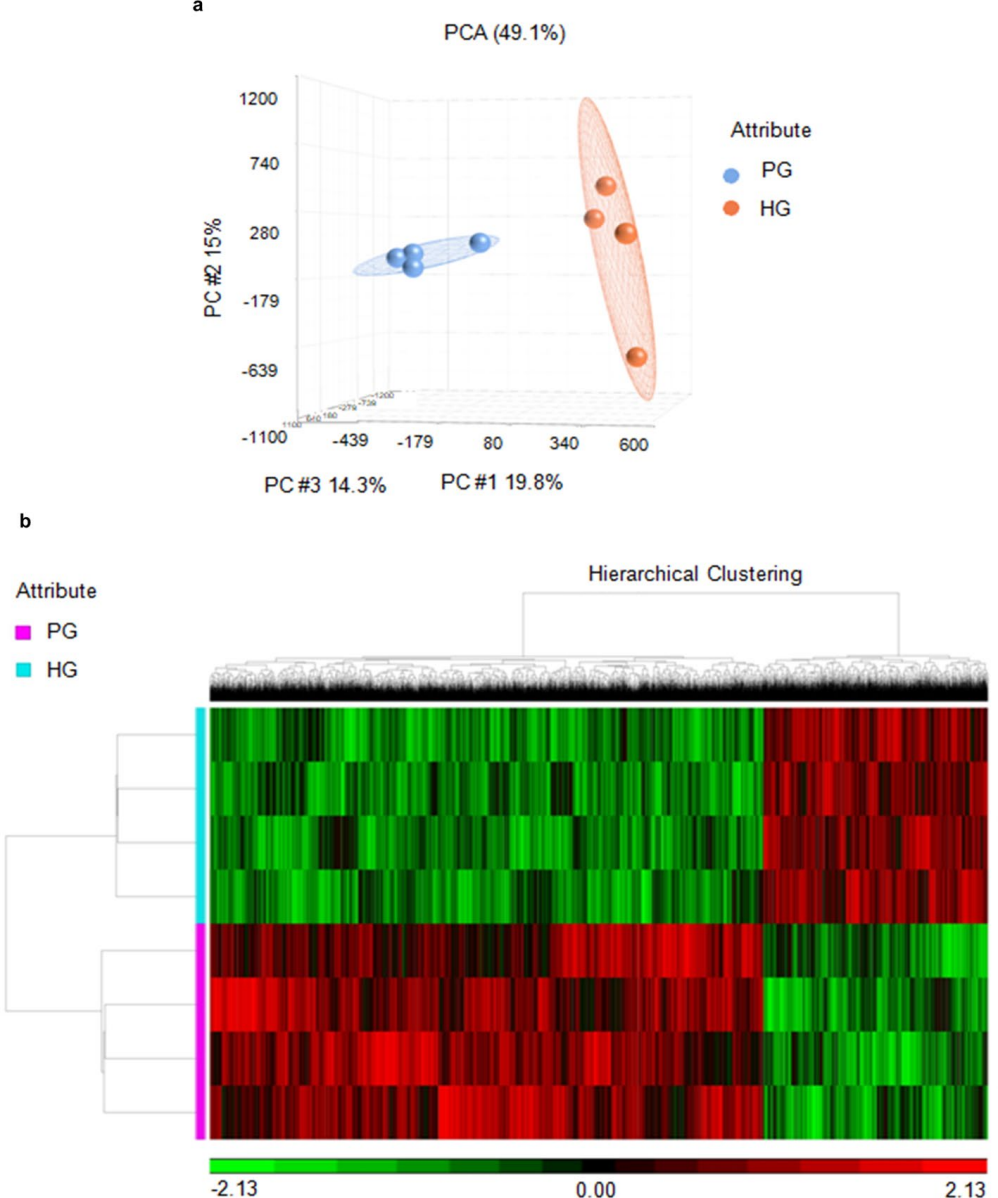
Whole genome DNA methylation profiling in hNPCs under PG or HG using Illumina Human MethylationEPIC array. (**a**) Principle component analysis (PCA) biplot of DNA methylation changes between hNPCs exposed to PG (Group1, blue) and HG (Group2, red), n = 4. (**b**) Representative heat-map depicting 12,087 probes which are differentially methylated in hNPCs exposed to PG (pink) and HG (cyan).

Epigenetic mechanisms have been shown to play a major role in regulating several molecular mechanisms responsible for the cell proliferation and lineage specification that occur during brain development^5,28^. In this study, DNA methylation has been the major focus, as it influences a variety of fundamental processes during brain development, such as neural stem cell renewal, cell fate specification, neuronal differentiation and maturation, as well as synaptogenesis^28,29^ by altering chromatin organization and gene expression. Hyper- or hypo-methylation of DNA during brain development has been associated with differentiation and lineage specification of NSCs^30,31^. In order to examine the gene specific DNA methylation status in hNPCs exposed to HG, a DNA methylation array (Illumina Human MethylationEPIC array) was performed in hNPCs exposed to PG or HG condition (Fig. 2). The raw data was Illumina normalized using Partek Genome suite version 6.6 and the principal component analysis revealed that the methylation pattern in hNPCs exposed to HG was clustered distinctly in comparison to that exposed to PG medium (Fig. 2a).

Next, the normalized data was subjected to one-way ANOVA and a list of filtered genes with 19,767 probes which passed the significant unadjusted *p*-value ≤ 0.05 and difference in the methylation levels between the PG and HG groups of hNPCs more than of ± 0.5 criteria, was created (Supplementary Dataset S1). Some miRNAs and L-ncRNAs with altered DNA methylation at parent sequence in the hNPCs exposed to HG condition were also found in the list of probes. Further, a heat map was generated on hierarchical clustering using top 12,087 differentially methylated probes in hNPCs exposed to HG (Fig. 2b). The gene ontology (GO) enrichment analysis from hNPCs exposed to HG based on cellular component, molecular function and biological processes revealed that: (a) genes involved in cellular junction, cytoskeleton and synapse were highly enriched in cellular component; (b) genes involved in binding and catalytic activity, including actin binding were enriched in molecular function; (c) genes involved in developmental process, axon guidance, metabolic processes and cell differentiation were highly enriched in biological processes (Supplementary Fig. S1, Supplementary Dataset S2). These findings indicate that HG alters DNA methylation pattern in genes regulating developmental processes in hNPCs. We have also used the *minfi* package^32^ to analyse the data (Supplementary Dataset S4) which were further subjected to pathway analysis using Ingenuity Pathway Analysis (IPA) software.

Lastly, in order to determine the signalling pathways affected by the HG in hNPCs, a pathway enrichment analysis was performed on differentially methylated genes in hNPCs exposed to HG using Partek Pathway suite and IPA. Both pathway analyses depicted that axon guidance pathway was one of the topmost affected pathway in hNPCs exposed to HG condition (Supplementary Dataset S3, S5). A number of genes from axon guidance pathway including SLIT1-ROBO2 pathway genes such as Slit guidance ligand 1(*SLIT1*), Roundabout guidance receptor 2 (*ROBO2*), SLIT-ROBO Rho GTPase activating protein 1 (*SRGAP1*) and Cell division cycle 42 (*CDC42*), which regulate axon guidance and brain patterning, was found to have altered methylation status in hNPCs exposed to HG. In addition, several genes including Yes-associated protein (*YAP*) and *WWTR1* (TAZ) from hippo signalling pathway were found to be differentially methylated in hNPCs exposed to HG.

### HG-induced hypomethylation at gene body in *SLIT1* was positively correlated with protein expression of SLIT1 in hNPCs

**Figure 3:**
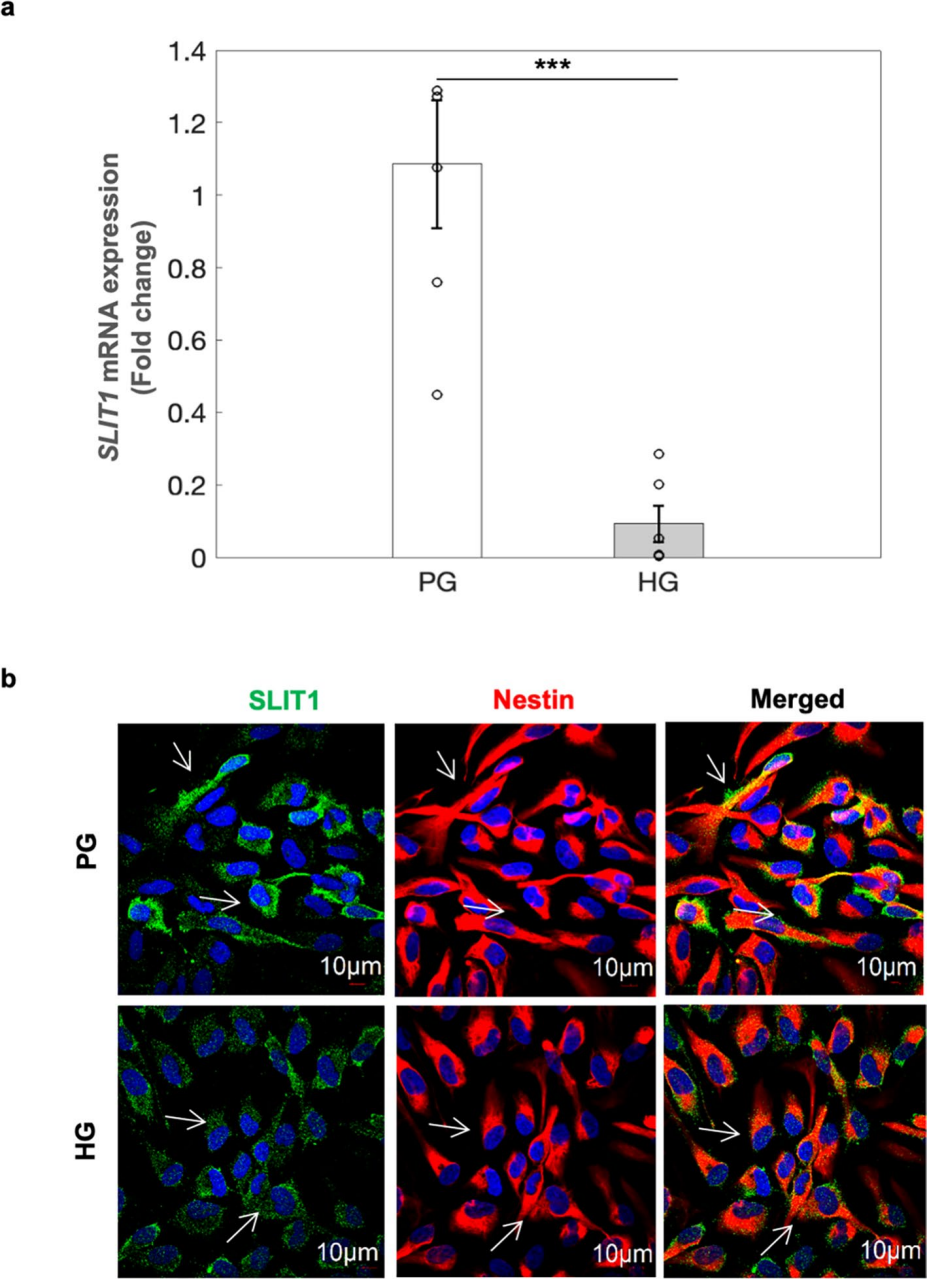
SLIT1 expression was decreased in hNPCs exposed to HG. (**a**) *SLIT1* mRNA expression was decreased in hNPCs exposed to HG condition by RT-PCR analysis. Data is represented as Mean ± SE (n = 6, ***p < 0.001). (**b**) Immunocytochemical analysis also revealed the decrease in SLIT1 expression (Green panel, indicated by arrows) hNPCs exposed to HG condition. Nestin is in Red while DAPI is blue (n = 3), scale bar = 10 μm.

**Figure 4:**
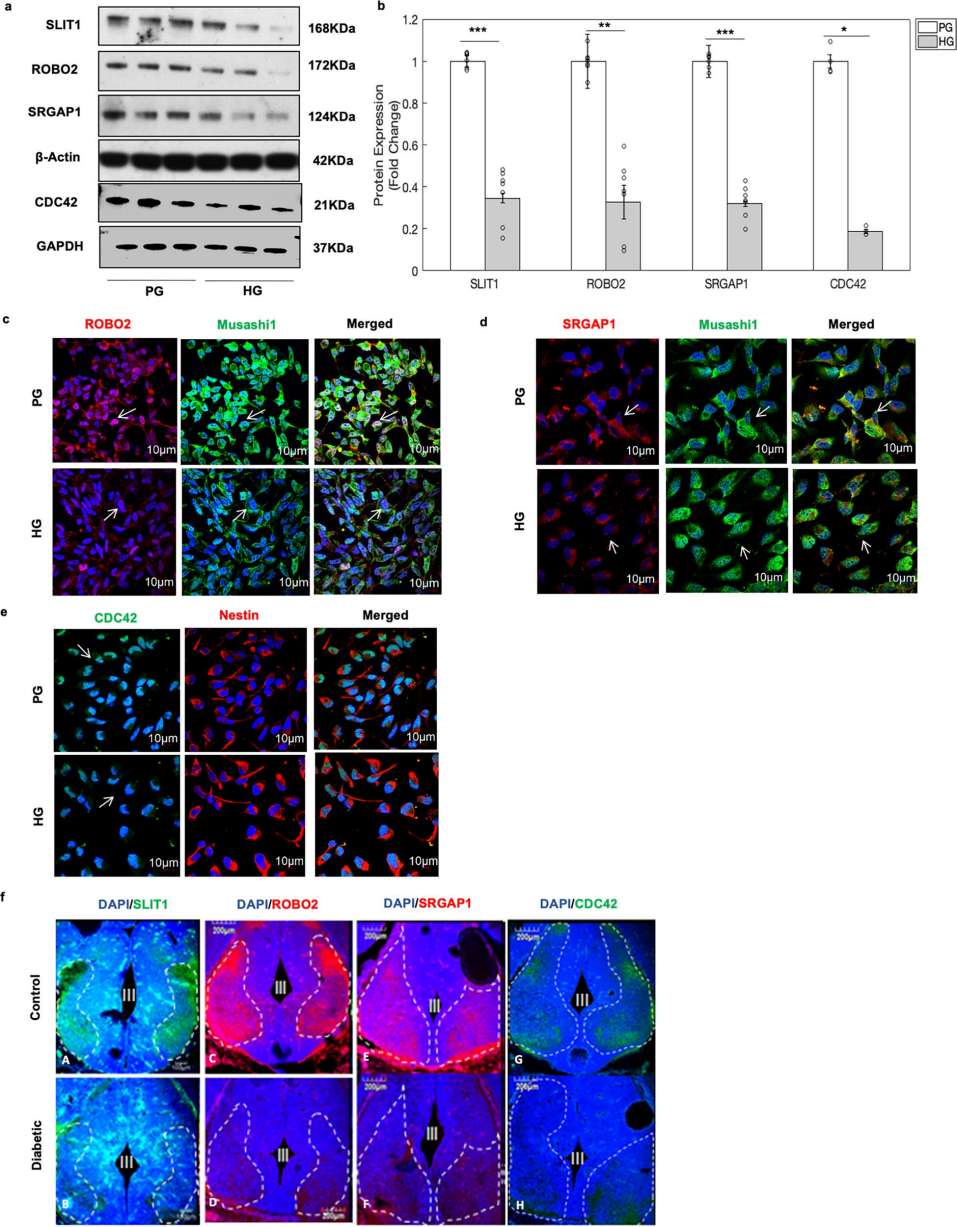
HG induced alterations in SLIT-ROBO signalling pathway genes. (**a**) Western blot analysis revealed the decreased protein expression levels of SLIT1, ROBO2, SRGAP1 and CDC42 in hNPCs exposed to HG. (**b**) Quantitative analysis depicts significant decrease in SLIT1 (168 kDa), ROBO2 (172 kDa), SRGAP1 (124 kDa) and CDC42 (21 kDa) protein expression in hNPCs exposed to HG condition. Data is represented as Mean ± SE, n = 3, *p < 0.05, **p < 0.01, ***p < 0.001. Uncropped blot images are shown in Supplementary Fig. S2. Further, the decrease in the expression of ROBO2 (**c**, Red), SRGAP1 (**d**, Red) and CDC42 (**e**, Green) in hNPCs was confirmed by immunocytochemical analysis. Musashi1 (Green) and Nestin (Red) was used to mark neural stem cells while DAPI was used to stain the nuclei (scale bar = 10 μm). (**f**) Furthermore, the protein expression of SLIT1 (**A**,**B**, Green), ROBO2 (**C**,**D**, Red), SRGAP1 (**E**,**F**, Red) and CDC42 (**G**,**H**, Green) were localised in forebrain sections of mouse embryos from normal and diabetic pregnancy. The expression levels of SLIT1, ROBO2, SRGAP1 and CDC42 appear to be markedly reduced in forebrain sections of mouse embryos diabetic pregnancy, compared to that of embryos from normal pregnancy, scale bar = 200 μm. The third ventricle is marked as III.

It has been shown that the DNA methylation at the gene body region has a positive correlation with its gene expression unlike DNA methylation at the promoter region of a gene which causes inverse relation with its gene expression^33^. As revealed by bioinformatic analysis, significant hypomethylation (6 of the 8 differentially methylated probes) was found in the gene body of *SLIT1* gene in hNPCs exposed to HG (Supplementary Dataset S6). Further validation by RT-PCR and western blot analyses showed a significant decrease in the expression levels of SLIT1 mRNA (Fig. 3a) and protein (Fig. 4a,b and uncropped blots in Supplementary Fig. S2) in hNPCs exposed to HG condition suggesting that HG-induced downregulation of SLIT1 expression in hNPCs, could be due to differential DNA methylation in *SLIT1* gene. Further, immunostaining analysis also showed marked decrease in expression of SLIT1 in hNPCs exposed to HG (Fig. 3b).

### HG epigenetically altered SLIT1-ROBO2 signalling pathway in hNPCs

**Figure 5:**
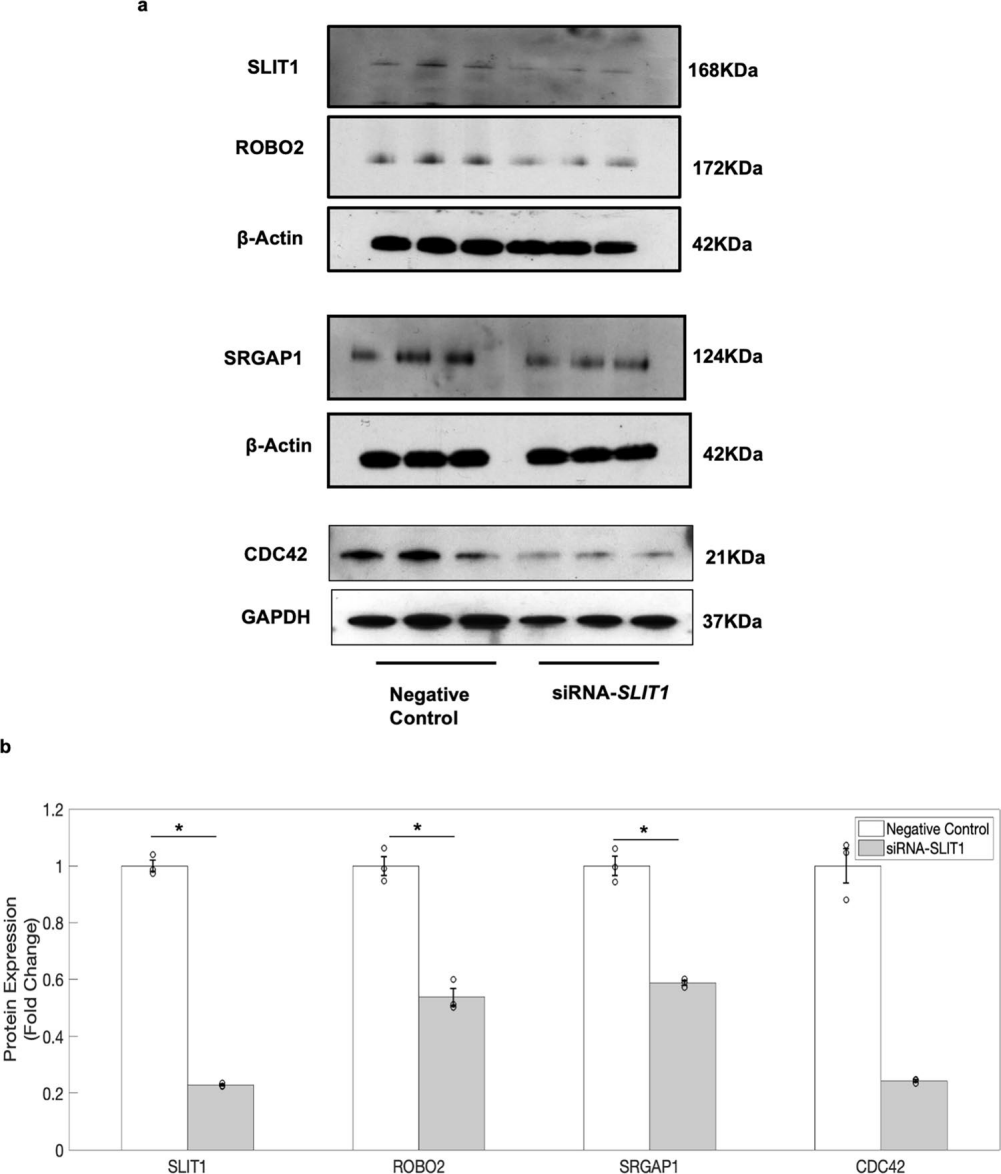
Downregulation of the SLIT-ROBO signalling pathway genes was confirmed in hNPCs following siRNA-mediated knockdown of *SLIT1*. (**a**) Western blot analysis showed that siRNA-mediated knockdown of *SLIT1* decreased the expression of ROBO2, SRGAP1, and CDC42 in hNPCs. (**b**) Quantitative analysis shows the reduction in the protein expression of SLIT1, ROBO2, SRGAP1 and CDC42 in hNPCs following siRNA-mediated knockdown of *SLIT1*. The data is represented as Mean ± SE, n = 3, *p < 0.05. Uncropped blot images are shown in Supplementary Fig. S2.

It is well known that the expression of a gene can be regulated by both molecular and epigenetic mechanisms^1^. Further, western blot analysis showed that the expression levels of SLIT1 downstream proteins i.e., ROBO2, SRGAP1 and CDC42 proteins were significantly decreased in hNPCs exposed to HG (Fig. 4a,b and uncropped blots in Supplementary Fig. S2). The decrease in the expression of ROBO2 (Fig. 4c), SRGAP1 (Fig. 4d), and CDC42 (Fig. 4e) in hNPCs exposed to HG was also confirmed by immunostaining analysis. Furthermore, immunohistochemical analysis on the forebrain sections of mouse embryos from diabetic pregnancy revealed a decrease in the expression of SLIT1 (Fig. 4f-B), ROBO2 (Fig. 4f-D), SRGAP1 (Fig. 4f-F) and CDC42 (Fig. 4f-H) proteins in comparison to that of control. Consistent with this, siRNA mediated knockdown of *SLIT1* in hNPCs (knockdown efficiency of 77.14%) downregulated the expression of ROBO2, SRGAP1, and CDC42 proteins (Fig. 5a,b and uncropped blots in Supplementary Fig. S2). Further, these *SLIT1* downstream genes, *ROBO2, SRGAP1* and *CDC42* were found to be differentially methylated in hNPCs exposed to HG (Supplementary Dataset S6). Majority of the differentially expressed probes were hypomethylated in the gene body of *ROBO2*, whereas differentially expressed probes associated with *CDC42* were found to be hypomethylated in 5′UTR and gene body. The differentially expressed probes associated with SRGAP1 were hypomethylated (6 of the 11 differentially methylated probes) in the gene body and hypermethylated (3 of the 11 differentially methylated probes) in the promoter. These findings suggest that the downregulation of SLIT1-ROBO2 signalling pathway genes in hNPCs exposed to HG could be due to interplay of both epigenetic and molecular mechanisms in hNPCs.

### Hippo signalling pathway was altered via SLIT1 in hNPCs exposed to HG

**Figure 6:**
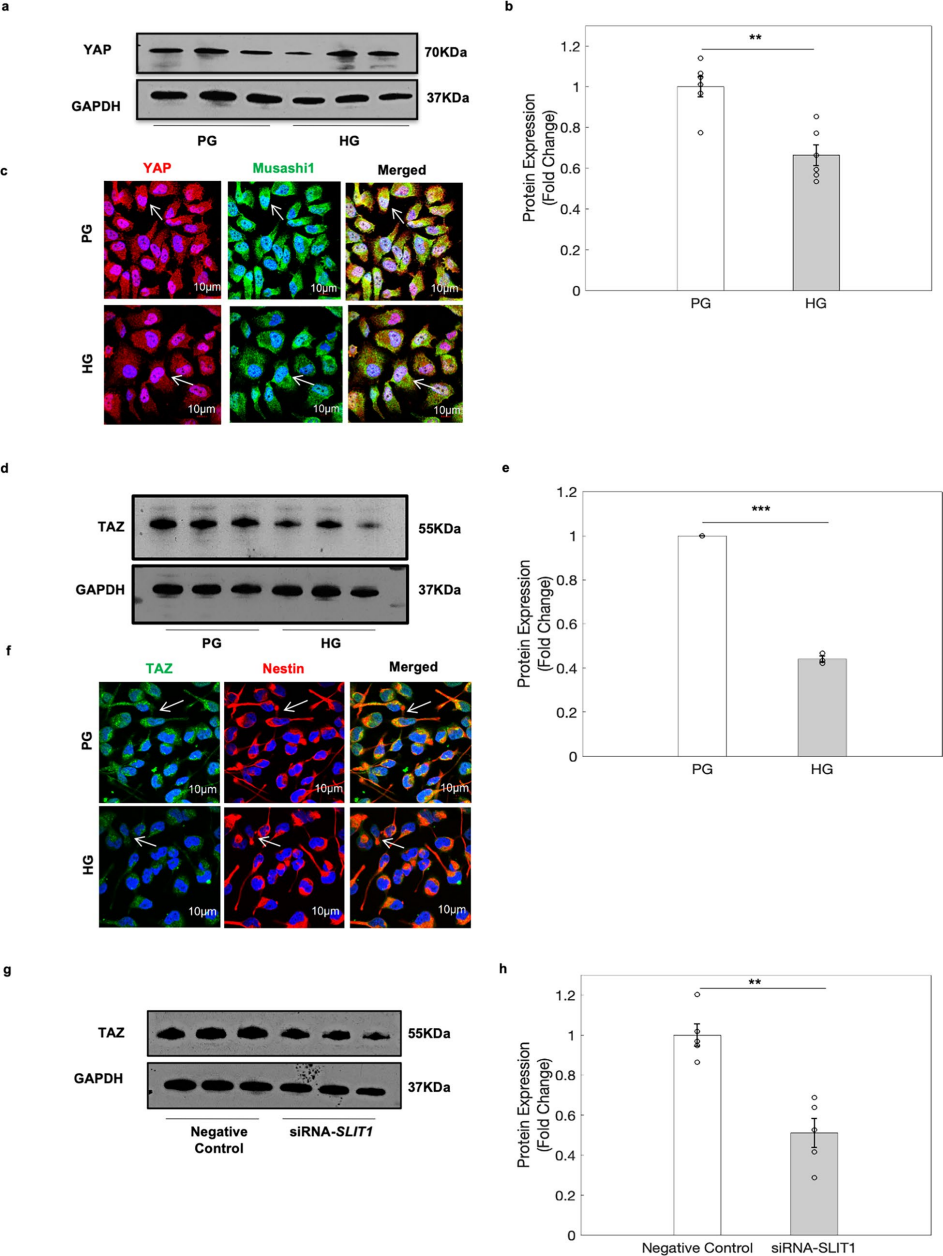
Hippo genes were significantly decreased in hNPCs under HG condition. (**a**) Western blot analysis revealed decrease in expression of YAP in hNPCs under HG condition. (**b**) Quantitative analysis shows significant decrease in YAP (70 kDa) protein expression in hNPCs exposed to HG condition. Data is represented as Mean ± SE, n = 3, *p < 0.05. (**c**) Immunocytochemical analysis confirmed the decrease in YAP (Red) expression in hNPCs exposed to HG. Musashi1 is in green while DAPI is in blue, scale bar = 30 μm. (**d**) Western blot analysis revealed decrease expression of TAZ in hNPCs exposed to HG condition. (**e**) Quantitative analysis shows significant decrease in TAZ (55 kDa) protein expression in hNPCs exposed to HG condition. Data is represented as Mean ± SE, n = 3, ***p < 0.001. (**f**) Immunocytochemical analysis confirmed the decrease in TAZ (Green) expression in hNPCs exposed to HG. Nestin is in red while DAPI is in blue, n = 3, scale bar = 10 μm. (**g**) Western blot analysis revealed significant decrease in TAZ expression in hNPCs following *SLIT1* knockdown. (**h**) Quantitative analysis shows significant decrease in TAZ expression in hNPCs following *SLIT1* knockdown. Data is represented as Mean ± SE, n = 3, **p < 0.01. Uncropped blot images are shown in Supplementary Fig. S2.

The hippo signalling pathway is well known for regulating organ growth and size^24^ via its effectors signalling, TAZ and its paralog, YAP^34^. Our data revealed that majority of the differentially expressed probes associated with *WWTR1*(TAZ) were hypomethylated in the 5′UTR and gene body. On the other hand, there was equal representation of both hyper- as well as hypomethylated probes associated with *YAP1* (Supplementary Dataset S6). Further, western blot analysis show that the expression levels of YAP (Fig. 6a,b and uncropped blots in Supplementary Fig. S2) and TAZ (Fig. 6d,e and uncropped blots in Supplementary Fig. S2) were found to be significantly downregulated in hNPCs exposed to HG condition. Immunostaining analysis further confirmed the decrease in the YAP (Fig. 6c) and TAZ (Fig. 6f) expression in hNPCs exposed to HG condition. Given that, SLIT1 regulates CDC42 which has been shown to interact with hippo signalling pathway^35^, the expression of TAZ was examined in hNPCs following knockdown of *SLIT1*^25^. The expression of TAZ was found to be significantly decreased after *SLIT1* knockdown in hNPCs (Fig. 6g,h and uncropped blots in Supplementary Fig. S2). The findings suggest that HG perturbs the epigenetic regulation of crosstalk between hippo signalling and axon guidance pathway through altered DNA methylation in hNPCs.

## Discussion

It is well established that hyperglycemia during pregnancy is a major risk factor for congenital malformations in various organ systems and cognitive impairments in offspring. Several studies have shown that maternal diabetes alters the expression of genes involved in neural tube development in mouse embryos^3,4^. Previously, we have shown that maternal diabetes alters the transcriptome in the cranial neural tube of mouse embryos and induces global alterations in epigenetic mechanisms such as DNA methylation, histone modifications and miRNA expression in the NSCs derived from the neural tubes of mouse embryos^3,4,9,16^. The present study sheds light on the underlying changes in gene-specific epigenetic regulatory pathways in hNPCs exposed to HG. Given that DNA methylation regulates the transcription network, it was demonstrated that high glucose alters the DNA methylation patterns of genes crucial for the development of normal brain structure and brain function.

The neural tube during development mainly consists of neural stem cells (NSCs), which are multipotent and self-renewing in nature. Upon differentiation stimuli, the NSCs give rise to neuronal and glial cell (astrocytes and oligodendrocytes) populations^36^. The NSCs undergo proliferation, migration, differentiation and cell fate specification to give rise to an appropriate number of neurons and glial cells for normal brain size and structure^5^. The differentiating neurons migrate to different levels of cortical layers with the assistance of guidance cues and radial glial cells (RGCs) which scaffold the whole process and form connections via synapses^37^. The whole neurulation process is regulated by epigenetic factors. During neurogenesis, dynamic DNA methylation changes occur in neural progenitor cells^38^. In mature neurons, DNA methylation plays an important role in synaptic plasticity associated with learning and memory^39^. Thus, any alterations in DNA methylation pattern in NSCs during development may cause premature differentiation leading to aberrant neurogenesis and synaptogenesis which may lead to abnormal brain patterning and function^40^. In the present study, altered DNA methylome in hNPCs exposed to HG appear to impair several signalling pathways involved in neural tube development.

During neural tube development, neuronal migration and instructive axonal growth are key processes guided by molecular cues such as Netrins, Semaphorins, Ephrins, and Slits^41–43^. SLIT1, a major axon guidance molecule, is expressed during early stages of cortical development and acts by binding with ROBO transmembrane receptors^19^. SLIT-ROBO signalling plays an important role in proliferation, migration, neurogenesis and axon guidance during brain development^18,20,21^. Alterations in SLIT-ROBO signalling may contribute to abnormal number and migration of neurons in developing brain regions, as well as aberrant axonal pathfinding which could affect brain structure, neuronal network and function^44–46^.

The present high-throughput DNA methylation analysis revealed that HG induced differential methylation of axon guidance pathway genes including *SLIT1,* and its downstream effectors, *ROBO2, SRGAP1* and *CDC42*^20,47–50^ in hNPCs. These results were associated with the down regulation of SLIT1, ROBO2, SRGAP1 and CDC42 proteins in hNPCs exposed to HG^33^. Though SLIT1 and its downstream targets in hNPCs were downregulated independently through altered DNA methylation by HG, it is possible that downregulation of SLIT1 could also contribute to downregulation of its downstream targets since the knockdown of *SLIT1* in hNPCs was found to reduce the expression of its downstream effectors, ROBO2, SRGAP1 and CDC42. This suggests that SLIT1-ROBO2 signalling pathway genes were perturbed via altered DNA methylation or *SLIT1* downregulation or both in hNPCs exposed to HG. Furthermore, impaired proliferation in hNPCs exposed to HG^4^ appears to be caused by the altered expression of SLIT-ROBO signalling genes which play important roles in proliferation, migration, and neurogenesis and axon guidance during brain development^18,20,21^.

Additionally, Hippo signalling pathway genes, YAP/TAZ have been implicated in neural progenitor cell proliferation, differentiation and neural tube development^24,51–53^. In the current study, the expression levels of both YAP and TAZ, which were differentially methylated, were found to be downregulated in hNPCs exposed to HG, suggesting that Hippo genes were dysregulated in hNPCs exposed to HG. A recent report showed that Hippo signalling pathway is regulated by CDC42^25^, which acts as an effector molecule for various cellular responses such as cell repulsion, actin dynamics and cell polarity, and is also implicated in axon guidance by cross-talk with other signalling pathways^48^. Furthermore, there was a significant decrease in the expression of TAZ in hNPCs following knockdown of *SLIT1*. Overall, this study demonstrates that Hippo gene *WWTR1*(TAZ) is (a) epigenetically regulated and (b) regulated by SLIT1-ROBO2 signalling pathway via CDC42 in hNPCs exposed to HG, suggesting a possible cross-talk between SLIT1-ROBO2 and Hippo signalling which was found to be perturbed in hNPCs exposed to HG.

**Figure 7:**
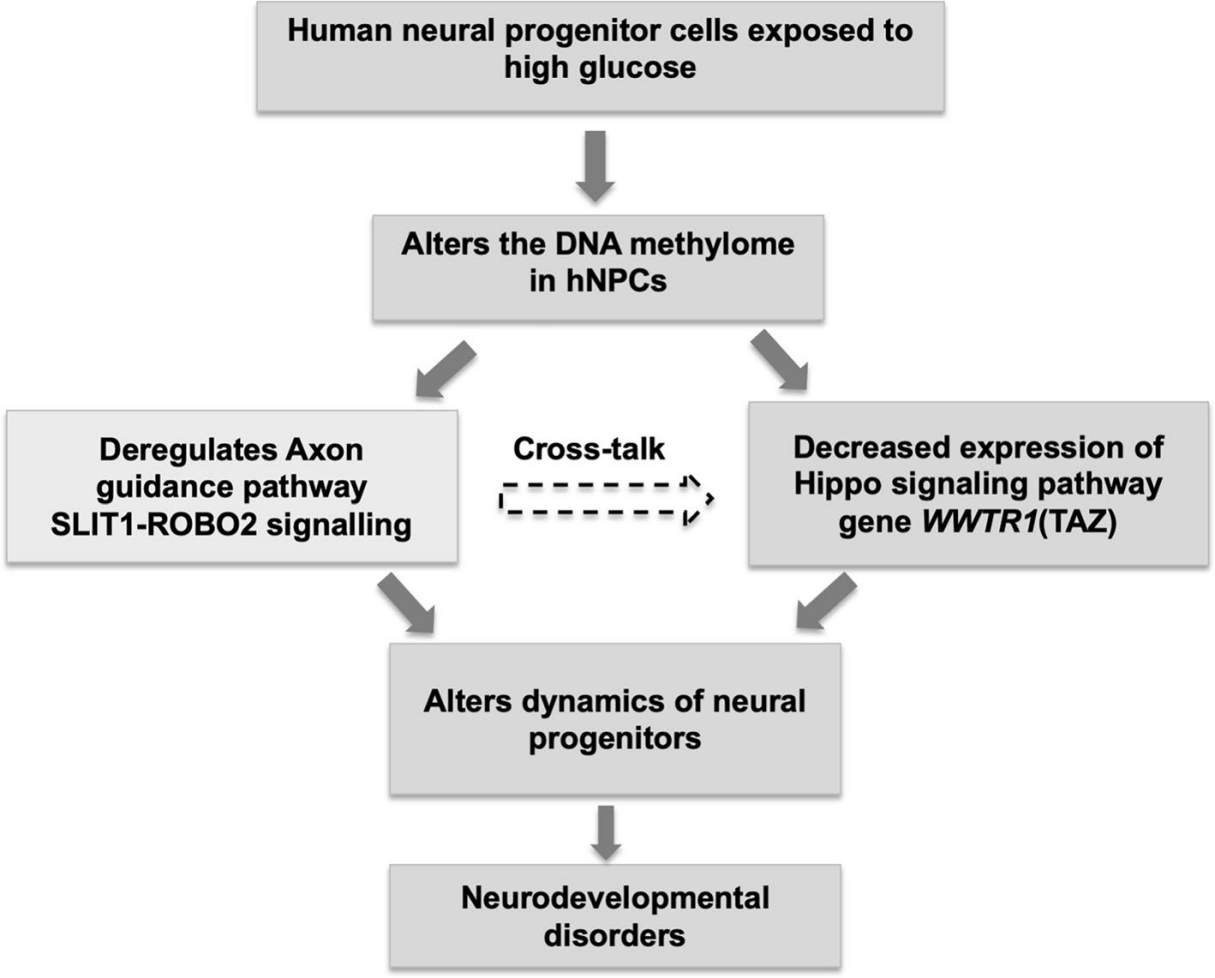
Overall summary. High glucose alters the DNA methylation status of key pathway genes such as axon guidance (*SLIT1-ROBO2*) and Hippo signalling (*WWTR1*) in hNPCs, forming the basis for neurodevelopmental disorders in offspring of diabetic pregnancy.

In conclusion, results from the present study demonstrate that exposure of hNPCs to high glucose alters the DNA methylation status of key pathway genes (axon guidance, Hippo signalling) which could form the basis for neurodevelopmental disorders observed in offspring of diabetic mothers (Fig. 7).

## Methods

### Cell lines and culture conditions

Human fetal neural progenitor cells (ReNcell VM) (Merck Millipore, Cat no. SCC008) were maintained at 37 °C and 5% CO_2_. All the culture dishes were coated with 10 μg/ml Laminin (Gibco, Cat. no. 1837951) for 4 h at 37 °C. Cells were maintained in DMEM, no glucose (Thermo Fisher Scientific, Cat. no. 11966025) and Ham’s F-12 Nutrient Mix, GlutaMAX Supplement (Thermo Fisher Scientific, Cat. no. 31765092) mixed in 1:1 ratio with supplements including 2% B-27 supplement (50X), serum free (Thermo Fisher Scientific, Cat. no. 17504001), 2 mM l-Glutamine (Thermo Fisher Scientific, Cat. no. 25030081), 1% Antibiotic–Antimycotic (Sigma Aldrich, Cat. no. A5955), 10 U/ml Heparin Sodium Cell Culture (Sigma Aldrich, Cat. no. H3149-50KU), 20 ng/ml Epidermal Growth Factor (EGF) recombinant human protein (Thermo Fisher Scientific, Cat. no. PHG0311) and 20 ng/ml fibroblast growth factor (Sigma Aldrich, Cat. no. F0291). Media was changed on every third day and cells were passaged when confluent.

### High glucose treatment

hNPCs were seeded at a seeding density of 0.05 × 10^6^ cells per well in a 6-well plate (Cellstar, Cat. no. 657160) in physiological glucose concentration (Day0). The next day (Day1), cells were replenished with media containing physiological glucose (5 mM, PG) or high glucose (40 mM, HG) (to mimic hyperglycemia in vitro), and the cells were cultured for 96 h (Day5) and used for various experiments.

### DNA extraction and quantification

DNA was extracted from the hNPCs using GeneJET Genomic DNA Purification Kit (Thermo Fisher Scientific, Cat. no. K0721) according to the manufacturer’s protocol. DNA was quantified using a Nanodrop (ND 1000).

### Whole genome DNA methylation profiling and data analysis

Whole genome DNA methylation profiling was performed using an Illumina Human MethylationEPIC array (Illumina, Cat. no. ILM-WG-317) with 850 K CpGs scan. All the samples were bisulphite converted using Zymo EZ DNA Methylation kit as per manufacturer’s protocol. The genomic DNA samples were subjected to bisulphite conversion in order to convert unmethylated cytosines into uracil. Following whole genome amplification, the uracil was replaced with thymine. The DNA was then enzymatically fragmented and applied to the Illumina Infinium MethylationEPIC array for hybridization. The methylated and unmethylated cytosines were identified using single base pair extension tagged with fluorophores. Then chip was scanned using Illumina iScan and the raw data was subjected to bioinformatic analysis. The output data were Illumina normalized and principal component analysis was done to extract the clusters from samples using Partek genome suite (Partek, Product Code: PGS_ACNL12R). Next, the normalized data was subjected to one-way ANOVA and a list of filtered genes with 19,767 probes which passed the significant unadjusted *p*-value ≤ 0.05 and difference in the methylation levels between the PG and HG groups of hNPCs more than of ± 0.5 criteria, was created. From these top 12,087 probes were used to generate the heatmap. Further, gene list was used to perform Gene Ontology based on cellular component, molecular function and biological process. In addition, pathway enrichment analysis was performed to observe most affected pathways using Partek Pathway suite (Partek, Product Code: PATH_ACNL12R). All steps were done using the online user tutorial for Partek software’s.

Additionally, the data was analysed using *minfi* package^32^. Briefly, the raw data was Illumina normalised and one-way ANOVA was performed. The data was filtered based on two criteria’s unadjusted p-value ≤ 0.05 and difference ± 0.5. About 29,663 probes passed the criteria’s (Supplementary Dataset S4). Furthermore, the gene list was subjected to core analysis using Ingenuity Pathway Analysis (Qiagen) and canonical pathways were obtained (Supplementary Dataset S5).

### RNA extraction and quantitative RT-PCR analysis

RNA was extracted with RNeasy Kit (Qiagen) as per the user guidelines. Total RNA was converted to cDNA by mixing 2 μg of RNA with 2 µl of OLigo(dT) (Invitrogen, Cat. no. 887748), followed by incubation at 70 °C for 5 min. Subsequently, a master mix comprising of 5 µl of M-MLV RT, 5X buffer (Invitrogen, Cat. no. 1890628), 1 μl of M-MLV reverse transcriptase, 0.7 µl of RNAse inhibitor (Invitrogen, Cat. no 1890636), and 0.5 μl of dNTP mix (Invitrogen, Cat. no. 1890631) was added to each tube. The reaction mix was topped up to 25 µl with water and samples were incubated at 42 °C for 1 h, 95 °C for 5 min. The cDNA was diluted 1:5 times and 1 µl of diluted cDNA was added to a mix of 5 µl of Fast SYBR green (Applied Biosystems) and 0.5 µl each of forward and reverse primer and the reaction mix was topped up to 10 μl with water. The quantitative PCR was performed in Applied Biosystems 7900HT Fast real-time PCR machine with fast protocol. The primers were designed using Primer-BLAST (NCBI) and the sequences of *SLIT1* primers are forward sequence: 5′-GGACTGTCGTGGAAAAGGC-3′ and reverse sequence: 5′-CCGTAGCTTTCTGTAGGGTGAG-3′. *β-actin* was used as control with forward sequence: 5′-CATGTACGTTGCTATCCAGGC-3′ and reverse sequence: 5′-CTCCTTAATGTCACGCACGAT-3′.

### Western blotting

Total protein was extracted using RIPA buffer (Thermo Fisher Scientific, Cat no. 89900) supplemented with 1X Halt Protease and Phosphatase Inhibitor Cocktail (Thermo Fisher Scientific, Cat. no. 78440) as per manufacturer’s protocol and protein samples were quantified using Bradford assay (Bio-Rad, Cat. no. 500-0006). 20-40 µg of the protein was denatured and loaded on 4–12% NuPAGE Bis–Tris Mini Gel (Invitrogen, Cat.no. NP0335BOX). Following electrophoresis, the separated proteins were transferred to nylon membrane by wet transfer method. The blots were blocked with 5% milk in PBS-Tx, following which the blots were incubated with rabbit anti-SLIT1 (1:100, ab129345, Abcam), mouse anti-ROBO2 (1:1,000, sc-376177, Santa Cruz Biotechnology), mouse anti-SRGAP1 (1:100, sc-81939, Santa Cruz Biotechnology), rabbit anti-YAP/TAZ (1:500, 8418S, Cell Signaling Technology), rabbit anti-CDC42 (1:50, #2462, Cell Signaling Technology), mouse anti-Nestin (1:500, ab18102, Abcam), rabbit anti-Musashi-1 (1:500, ab52865, Abcam), mouse anti-GAPDH (1:1,000, G8795, Sigma Aldrich) or mouse β-actin(1:5,000, A1978, Sigma Aldrich) prepared in 3% milk in PBS-Tx overnight at 4 °C. Subsequently, appropriate secondary peroxidase-conjugated antibody was prepared in 3% milk in PBS-Tx and the blots were incubated for 1 h at room temperature. The target proteins were visualized using chemiluminescent substrate (Pierce) and bands were developed on X-ray films. Equal protein loading was confirmed by stripping the blots and incubating with β-actin or GAPDH antibody. The protein bands were quantitated using GS-800 densitometer (Bio-Rad). All the uncropped blot images are shown in Supplementary Fig. S2.

### Immunocytochemistry

hNPCs were cultured on laminin-coated 13 mm glass coverslips, and the cells were fixed post HG treatment using 4% paraformaldehyde (PFA) for 18 min at room temperature, and then blocked with 5% goat serum for 1 h. Subsequently, the cells were incubated at 4 °C overnight with rabbit anti-SLIT1 (1:100, ab129345, Abcam), mouse anti-ROBO2 (1:200, sc-376177, Santa Cruz Biotechnology), mouse anti-SRGAP1 (1:200, sc-81939, Santa Cruz Biotechnology), mouse anti-YAP (1:50, sc-101199, Santa Cruz Biotechnology), rabbit anti- TAZ (1:50, ab84927, Abcam), rabbit anti-CDC42 (1:50, #2462, Cell Signaling Technology), mouse anti-Nestin (1:250, ab18102, Abcam), mouse anti-SOX-2 (1:250, ab171380, Abcam) or rabbit anti-Musashi-1 (1:500, ab52865, Abcam). The following day, the coverslips were incubated with secondary antibody for 1 h at room temperature. The nucleus was counterstained with 4′, 6-diamidino-2-phenylindole (DAPI) for 5 min, and the coverslips were mounted with fluorescence mounting media (DAKO, Cat. no. S3023) onto glass slides. Images were taken using a confocal microscope, LSM FV1000 (Olympus).

### MTS assay

hNPCs were grown in 24 well plates at a seeding density of 15,000 cells per well and exposed to PG or HG conditions for 96 h. Post-treatment the hNPCs were incubated with 1:5 diluted MTS solution (CellTiter, 96 Aqueous) in 1:1 DMEM (no glucose) and F12, Ham’s supplement media for 45 min at 37 °C, and the absorbance was measured at 495 nm using a spectrophotometer (Tecan F200).

### siRNA transfection

hNPCs were seeded in 6 well plates at a density of 2.5 × 10^5^ cells/well. 24 h post- seeding, the hNPCs were transfected with ON-TARGETplus Non-targeting Control Pool (Dharmacon, D-001810-01-05) or ON-TARGETplus human *SLIT1* siRNA (L-012599-00-0005, Dharmacon) using Lipofectamine RNAiMAX (Life Technologies), following the manufacturer’s instructions. The complexes were prepared in Opti-MEM (Gibco, Cat. no. 31985-070) and added to the cells at a final concentration of 30 nM and cells were harvested 72 h post-transfection.

### Collection of embryos from pregnant mice

Animals were purchased from Centre for animal resources (CARE, NUS). The animal handling and procedures used in the current study were approved by Institutional Animal Care and Use Committee (IACUC), National University of Singapore (Protocol number: R15-0030). All methods were performed in accordance with the relevant guidelines and regulations. The diabetic embryos were obtained as described previously^4,16^. A total of 12 mice (4 mice for normal pregnancy, 4 mice for diabetic pregnancy and 4 male mice for mating) were used in this study. Briefly, diabetes was induced in 8-week old Swiss albino female mice by an intra-peritoneal injection of Streptozotocin solution (STZ prepared in 0.01 M citrate buffer with pH 4.5, 75 mg/kg body weight). After 1 week, the mice were screened for diabetes with criteria of non-fasting blood glucose levels > 200 mg/dl. Diabetic female mice were mated with a healthy age matched male mice overnight (3–4 diabetic females/healthy male within one cage). The day when the vaginal plug was seen was counted as embryonic day 0.5 (E 0.5). Age matched time mated pregnant mice were purchased from CARE, NUS and used as the control. The embryos were collected on E13.5 by anesthetising pregnant females from normal or diabetic groups with pentobarbital (150 mg/kg body weight) followed by caesarean section. The embryos were collected and placed in PBS solution. The embryonic brains were fixed by placing first in 4% PFA overnight and then transferred into 30% sucrose for 24 h. The tissue specimen was trimmed and oriented before mounting on Shandon embedding matrix using liquid nitrogen. The sections are cut using cryostat equipment with appropriate thickness and transferred to pre-coated gelatin slides. The sections were stored in − 20 °C for long term use.

### Immunohistochemistry

The fixed mouse sections were permeabilized with 0.1% PBS-Tx for 10 min, following which sections were washed twice with PBS for 10 min each. The sections were then blocked with 5% goat serum for 1 h. Next, the sections were incubated with rabbit anti-SLIT1 (1:25, ab129345, Abcam), mouse anti-ROBO2 (1:100, sc-376177, Santa Cruz Biotechnology), mouse anti-SRGAP1 (1:50, sc-81939, Santa Cruz Biotechnology) or rabbit anti-CDC42 (1:25, #2462, Cell Signaling Technology), overnight. The following day, the sections were washed with PBS thrice for 10 min each before incubation with secondary antibody for 1 h. Next, the sections were washed thrice with PBS thrice for 10 min each, and the nucleus was counterstained with DAPI. The coverslips were mounted using mounting medium (DAKO) and imaged using a confocal microscope (Olympus FV1000).

### Statistical analysis

At least two independent experiments were performed using 4–6 samples, each of control or experimental group of hNPCs. The data are represented as mean ± SEM and student’s *t*-test was done using Microsoft excel. The results were considered significant when *p* < 0.05.

## Supplementary information

Supplementary Figures

Supplementary Dataset S1.

Supplementary Dataset S2.

Supplementary Dataset S3.

Supplementary Dataset S4.

Supplementary Dataset S5.

Supplementary Dataset S6.

## Data Availability

All data generated or analysed during this study are included in this published article (and its Supplementary Information files).
