# Supplementary material for: High glucose alters the DNA methylation pattern of neurodevelopment associated genes in human neural progenitor cells in vitro
Source: Sci Rep. 2020 Sep 24;10:15676. doi: 10.1038/s41598-020-72485-7 (PMC7518427; doi:10.1038/s41598-020-72485-7)
Supplement: Supplementary file 1 — Supplementary Figures [file 41598_2020_72485_MOESM1_ESM.pdf]

Supplementary Data

High glucose alters the DNA methylation pattern of neurodevelopment associated genes in human neural progenitor cells *in vitro*

Deepika Kandilya[1], *Sukanya Shyamasundar*[1], *Dhiraj Kumar Singh*[1], *Avijit Banik*[1], *M. Prakash Hande*[2], *Walter Stünkel*[4], *Yap Seng Chong*[3], *S. Thameem Dheen*[1*]

[1]*Department of Anatomy,* [2]*Department of Physiology,* [3]*Department of Obstetrics and Gynaecology, Yong Loo Lin School of Medicine, National University of Singapore*

[4] *Present address: Experimental Drug Development Centre, Agency for Science, Technology and Research, Singapore*

****Corresponding Author: Associate professor S. Thameem Dheen****; Email: antstd@nus.edu.sg; ORCID iD:* 0000-0001-9600-6789; *Tel.no.: +65-65163200; Address: Department of Anatomy, Yong Loo Lin School of Medicine, National University of Singapore,4 Medical Drive, MD10, Level4, Singapore 117594*

Supplementary Figure: 1

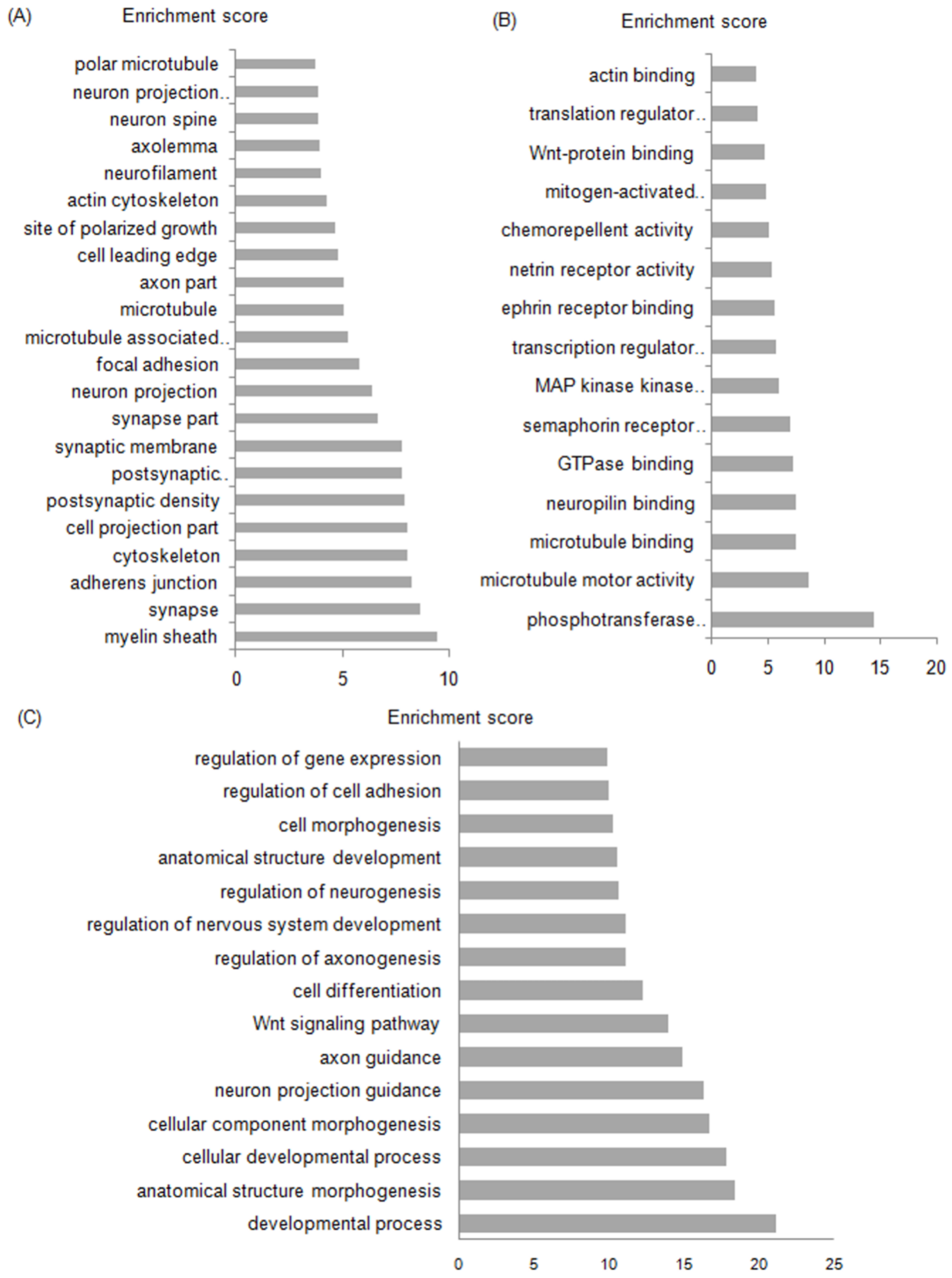

Supplementary Figure 1: Gene ontology based on cellular component (a), molecular function (b), and biological processes (c).

Supplementary Figure : 2

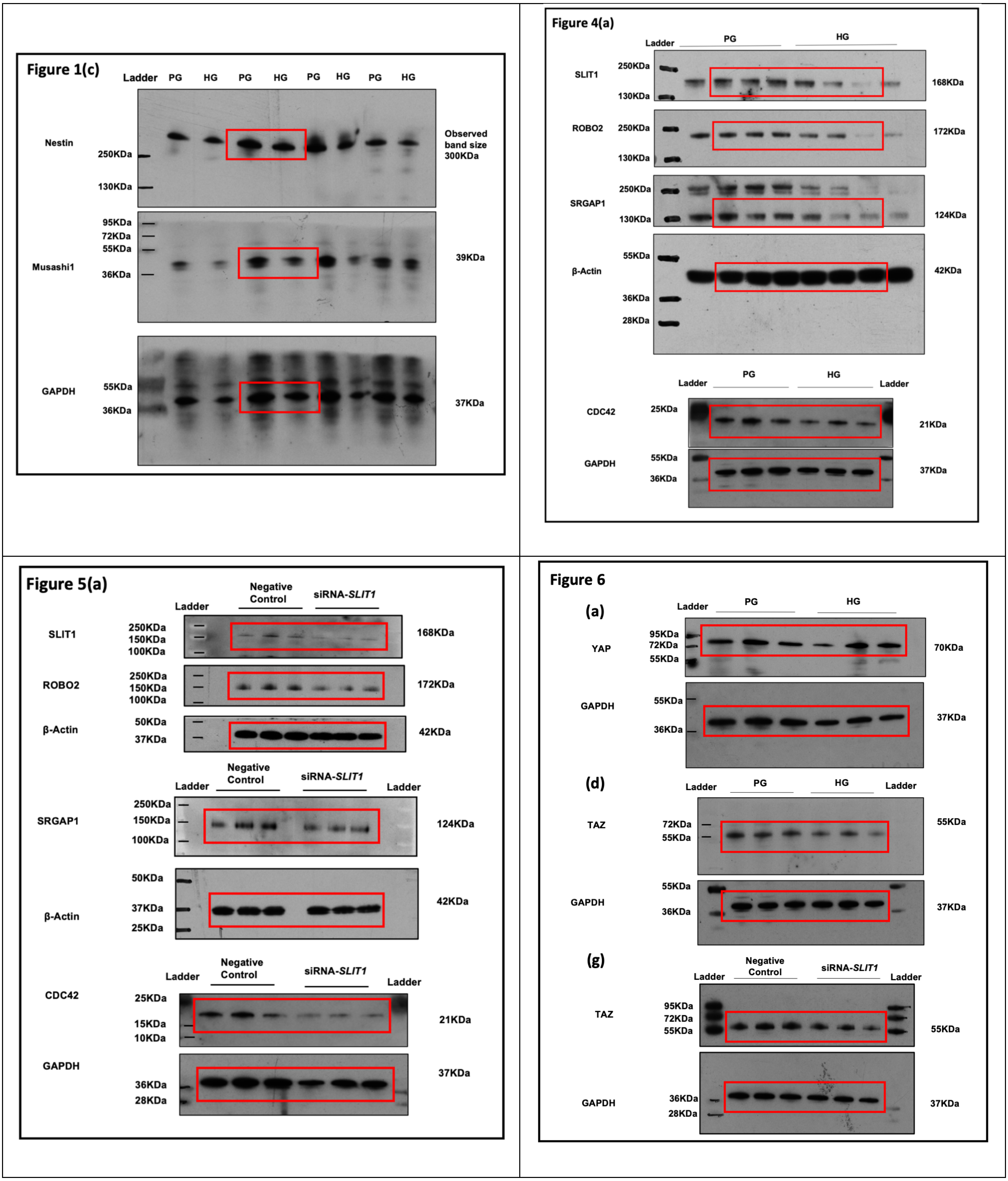

Supplementary Figure 2: Uncropped Western blots, shown in Figure 1c, 4a, 5a, and 6a, d and g. Cropped areas are highlighted by red box.
